# Supplementary material for: Local pH oscillations witness autocatalytic self-organization of biomorphic nanostructures
Source: Nat Commun. 2017 Feb 16;8:14427. doi: 10.1038/ncomms14427 (PMC5316880; doi:10.1038/ncomms14427)
Supplement: Supplementary Information — Supplementary Figures [file ncomms14427-s1.pdf]

Supplementary Figures

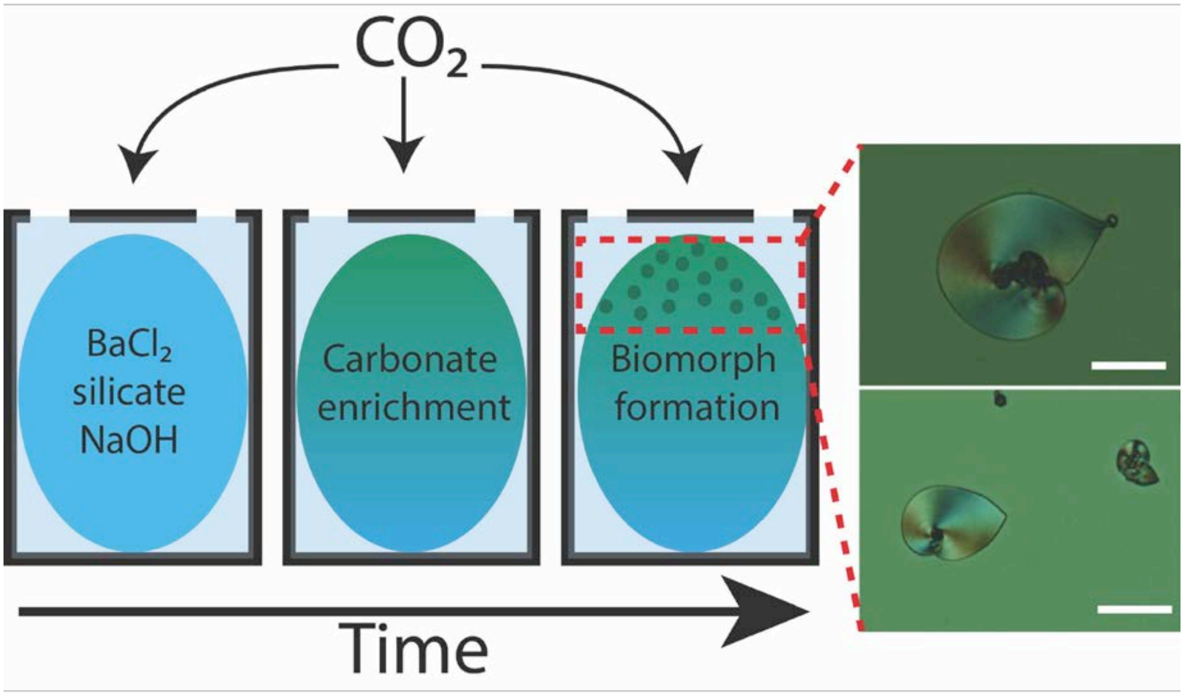

Supplementary Figure 1. Schematic drawing of the experimental setup used for the growth of silica-barium carbonate biomorphs in thin cells suitable for fluorescence microscopy. A drop of an alkaline solution containing barium ions and silicate species was placed between a glass slide and a coverslip that were spaced by adhesive tape (black lines). Atmospheric carbon dioxide then diffuses into the solution and ultimately induces carbonate precipitation and the formation of biomorphs (dark green spots), preferentially near the two holes on the top of the cell. The polarized optical micrographs on the right show typical biomorphic structures (rounded sheets) obtained under these conditions. Scale bar 50 μm.

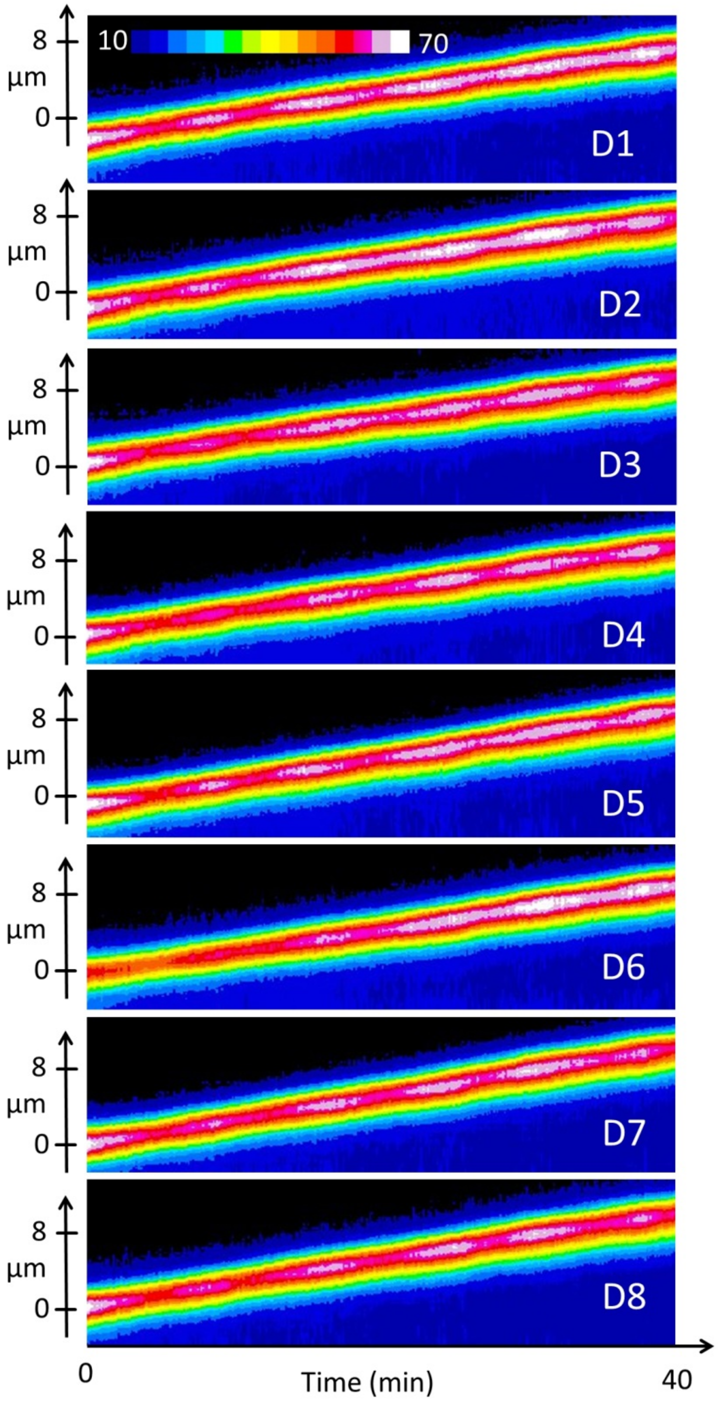

Supplementary Figure 2. Contour maps of the fluorescence intensity distribution along the growth vectors D1-D8 (defined in Fig. 2c of the main text) as a function of time.

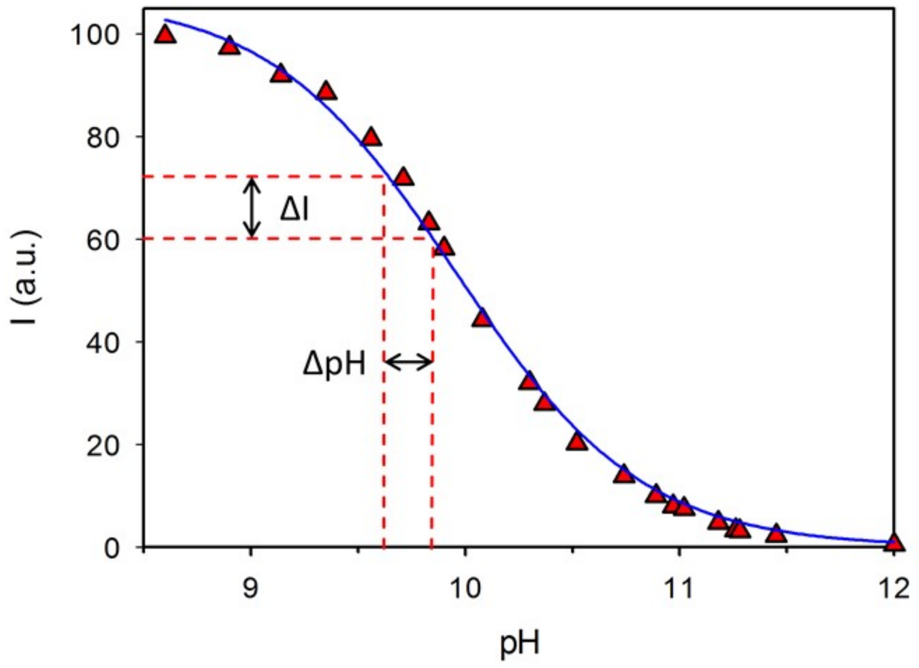

Supplementary Figure 3. Plot of the fluorescence intensities measured for 10^{-5} M aqueous solutions of AO at different predefined pH values. The experimental data (red triangles) were fitted using Equation 1 in Methods, section Fluorescence Microscopy, to give the blue line. The range of fluorescence intensities measured at the local fluorescence peak during growth of silica biomorphs is shown as ΔI together with the corresponding estimated local pH levels (ΔpH).
